# Ultrasound assessment of lymph nodes for staging of gynecological cancer: consensus opinion on terminology and examination technique

**DOI:** 10.1002/uog.29127

**Published:** 2024-11-08

**Authors:** D. Fischerova, E. Gatti, C. Culcasi, Z. Ng, G. Szabó, L. Zanchi, A. Burgetova, O. Nanka, G. Gambino, M. R. Kadajari, G. Garganese, Collaborators, N. Tiszlavicz, F. Sousa

**Affiliations:** ^1^ Department of Gynecology, Obstetrics and Neonatology, First Faculty of Medicine Charles University and General University Hospital in Prague Prague Czech Republic; ^2^ Department of Biomedical Science for Health University of Milan Milan Italy; ^3^ Department of Woman and Child Health and Public Health, Fondazione Policlinico Universitario Agostino Gemelli IRCCS Università Cattolica del Sacro Cuore Rome Italy; ^4^ Department of Gynaecological Oncology KK Women's and Children's Hospital Singapore; ^5^ Department of Obstetrics and Gynaecology, Faculty of Medicine Semmelweis University Budapest Hungary; ^6^ Department of Clinical, Surgical, Diagnostic and Pediatric Sciences, Unit of Obstetrics and Gynaecology University of Pavia, IRCCS San Matteo Hospital Foundation Pavia Italy; ^7^ Department of Radiology, First Faculty of Medicine Charles University and General University Hospital in Prague Prague Czech Republic; ^8^ Institute of Anatomy, First Faculty of Medicine Charles University Prague Czech Republic; ^9^ Department of Gynecologic Oncology, ARNAS Civico Di Cristina Benfratelli University of Palermo Palermo Italy; ^10^ Obstetrics and Gynaecology Department University Hospital Waterford Waterford Ireland; ^11^ Unità Operativa di Chirurgia degli Organi Genitali Esterni Femminili, Divisione di Ginecologia Oncologica, Dipartimento Scienze della Salute della Donna, del Bambino e di Sanità Pubblica Fondazione Policlinico Universitario A. Gemelli IRCCS Rome Italy; ^12^ Gemelli Woman Health Center for Digital and Personalized Medicine, Dipartimento Scienze della Vita e Sanità Pubblica Università Cattolica del Sacro Cuore Rome Italy

**Keywords:** anatomy, clinical cases, gynecological malignancy, lymph nodes, lymphatic drainage, ultrasonography

## Abstract

The lymphatic pathway is an important route of metastasis in gynecological malignancy. Therefore, the examination of lymph nodes is an essential part of the ultrasound evaluation in patients with known or suspected gynecological malignancy. The lymph nodes most frequently involved in gynecological malignancy (apart from vulvar cancer) are parietal (retroperitoneal) and visceral abdominopelvic lymph nodes. In advanced disease, more distant lymph‐node regions, such as the inguinal, axillary and supraclavicular lymph nodes, can also be involved. The standardized description of lymph nodes has been published previously by the Vulvar International Tumor Analysis (VITA) collaborative group. Herein, a collaborative group of gynecologists and gynecological oncologists with extensive ultrasound experience presents a systematic methodology for ultrasonographic lymph‐node assessment performed as part of the locoregional and distant work‐up to assess the extent of gynecological malignancy. The aim of this consensus opinion is also to describe the anatomical classification and drainage pathways of the lymphatic system as relevant to the gynecological organs. © 2024 The Author(s). *Ultrasound in Obstetrics & Gynecology* published by John Wiley & Sons Ltd on behalf of International Society of Ultrasound in Obstetrics and Gynecology.

## INTRODUCTION

**Figure 1:**
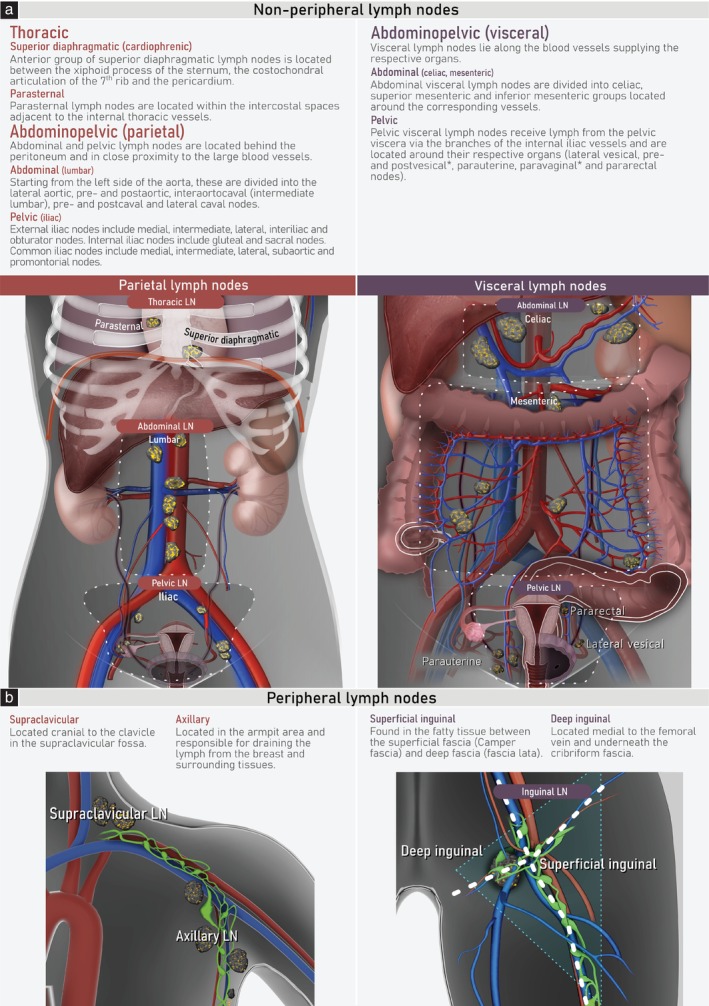
Schematic diagram showing lymph‐node (LN) classification. The image shows non‐peripheral (a) and peripheral (b) lymph nodes, which can be infiltrated by gynecological cancers. *Not visible in coronal view.

Lymph nodes are classified based on their location and can be divided into two main categories: peripheral and non‐peripheral lymph nodes (Figure 1). Non‐peripheral lymph nodes may be further subdivided into parietal lymph nodes, including thoracic, abdominal (lumbar) and pelvic (iliac) nodes, and visceral lymph nodes, namely the abdominal visceral and pelvic visceral lymph nodes. Peripheral nodes include the supraclavicular, axillary and inguinal lymph nodes.

**Figure 2:**
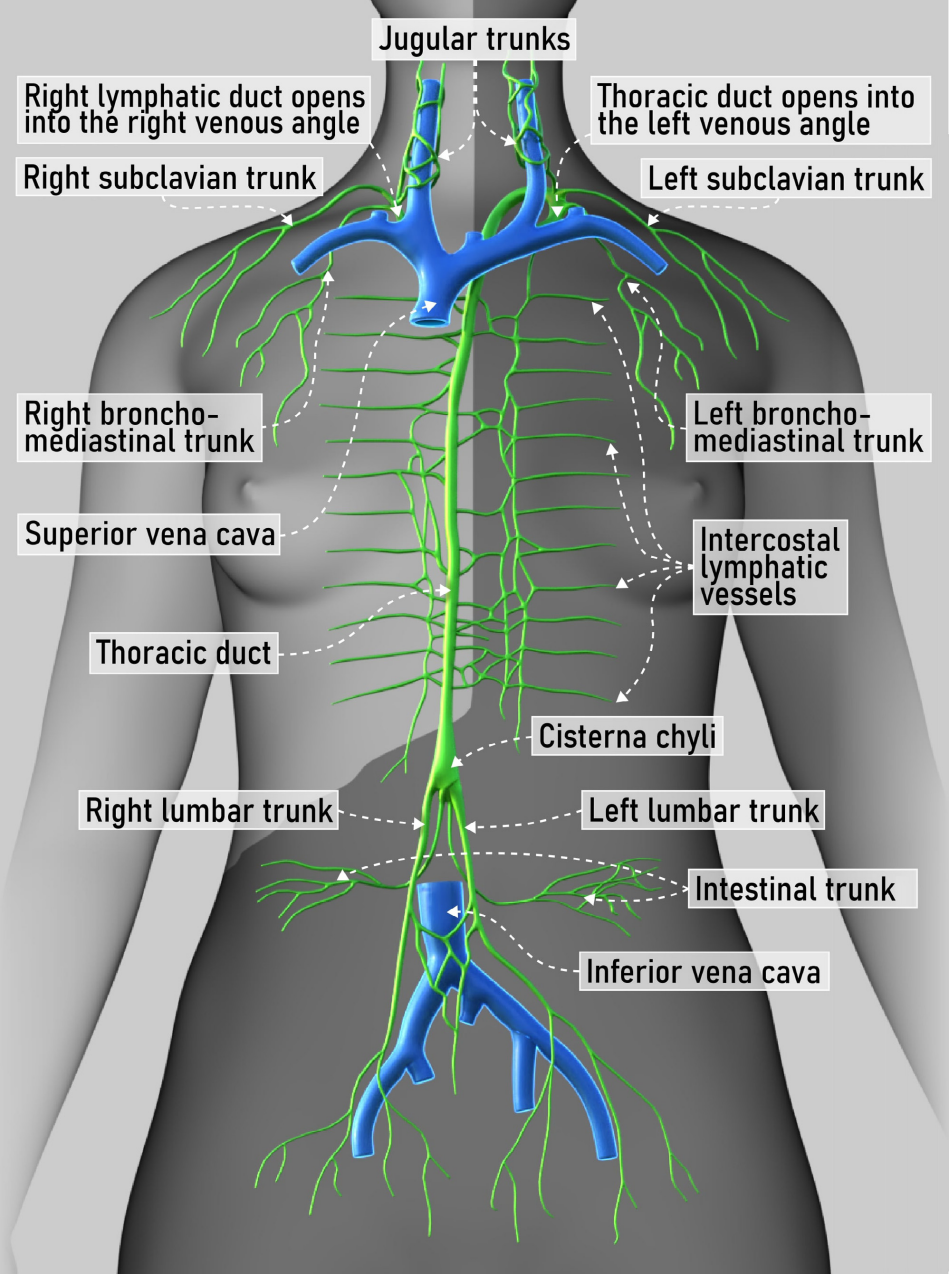
Schematic diagram showing major lymph vessels of the body. In the abdomen, the lumbar trunks are formed by merging of the efferent vessels from the abdominal lumbar lymph nodes; the intestinal trunk receives lymph from the celiac, superior mesenteric and inferior mesenteric lymph nodes (abdominal visceral). These trunks merge to form the cisterna chyli at the level of the first and second lumbar vertebrae, which continues as the thoracic duct in its cranial portion and finally empties at the level of the left jugulosubclavian venous angle, converging with left jugular, subclavian and bronchomediastinal trunks. The right lymphatic duct is formed by the right bronchomediastinal, jugular and subclavian lymphatic trunks and opens into the right jugulosubclavian venous angle. The dark grey region represents the drainage of the thoracic duct (three‐quarters of the body), and the light grey region represents the drainage of the right lymphatic duct (one‐quarter of the body).

The lymphatic pathway is an important route of metastasis in gynecological malignancy. Lymphatic fluid from the pelvic reproductive organs drains mostly via the pelvic (internal and external iliac) lymph nodes and subsequently to the common iliac lymph nodes. However, the ovary and the Fallopian tube can also drain directly into the abdominal lumbar nodes via lymphatic channels lying alongside the ovarian vessels, and endometrial cancer can sometimes also exhibit isolated involvement of abdominal lumbar nodes due to lymphatic spread via the same pathway^1^. In contrast, vulvar and lower vaginal cancers most frequently involve the inguinal lymph nodes. Infradiaphragmatic and supradiaphragmatic lymph nodes are connected by a complex network of lymphatic vessels. From the common iliac lymph nodes, lymphatic fluid travels to reach the left and right lumbar lymphatic trunks, which merge with the intestinal lymphatic trunk to form the cisterna chyli and subsequently the thoracic duct, entering the venous circulation at the left venous angle between the left subclavian and internal jugular veins (Figure 2)^2^. The thoracic duct collects 75% of the lymph of the entire body, representing the main lymphatic drainage system. The right lymphatic duct collects lymph from the upper right side of the body. It drains into the venous system at the junction formed by the convergence of the right internal jugular vein and the subclavian (or brachiocephalic) vein.

Traditional imaging modalities for assessment of lymphatic spread include contrast‐enhanced computed tomography, magnetic resonance imaging and fluorine‐18‐fluorodeoxyglucose positron emission tomography/computed tomography (FDG‐PET‐CT). In recent years, improved ultrasound technology and increased ultrasound resolution have enabled detailed sonographic assessment of even minute changes in lymph‐node architecture. This is especially true if the ultrasound transducer can be placed in close proximity to the lymph‐node region to be examined, such as using a transvaginal or transrectal approach to assess the pelvic lymph nodes, or a transcutaneous approach to assess the peripheral lymph nodes. These technological improvements, combined with its safety, cost‐effectiveness and patient tolerance, have meant that ultrasound has become an effective technique for lymph‐node assessment^3^, ^4^.

In 2021, the Vulvar International Tumor Analysis (VITA) group produced a consensus opinion on the ultrasound methodology of inguinal lymph‐node evaluation and standardized terminology for lymph‐node description in vulvar cancer staging (Figure S1 and Table S1)^5^. Based on the evaluation of lymph‐node morphology and vascular pattern, changes possibly related to metastatic involvement can be identified. Although the proposed terminology was created in the context of vulvar cancer, it can be applied to lymph‐node morphology and perfusion anywhere in the body if adequate acoustic resolution can be achieved, and may help in differentiating between infiltrated and non‐infiltrated (normal, reactive and postreactive) lymph nodes.

The role of ultrasound in the preoperative work‐up of gynecological cancer, based on updated European guidelines, was summarized in a recent review article^6^. Systematic abdominopelvic and groin ultrasound imaging, following a predefined checklist for each type of gynecological cancer, aims to classify the anatomical extent of the spread of malignant tumors, according to the TNM classification (T, tumor; N, regional lymph node; M, distant metastases), taking into account all additional prognostic parameters, to enable individualized treatment. The purpose of this consensus opinion is to provide a methodological guide to allow a structured, systematic approach towards assessment of the lymph nodes as part of gynecological cancer staging. It presents the following groups of lymph nodes, exploring their anatomical classification^7^ and the ultrasound examination technique for each, supported by schematic diagrams and videoclips: (1) infradiaphragmatic lymph nodes (parietal (retroperitoneal), visceral and inguinal lymph nodes); and (2) supradiaphragmatic lymph nodes (superior diaphragmatic (cardiophrenic), supraclavicular, axillary and parasternal (internal mammary/thoracic) lymph nodes).

## INFRADIAPHRAGMATIC LYMPH NODES

**Figure 3:**
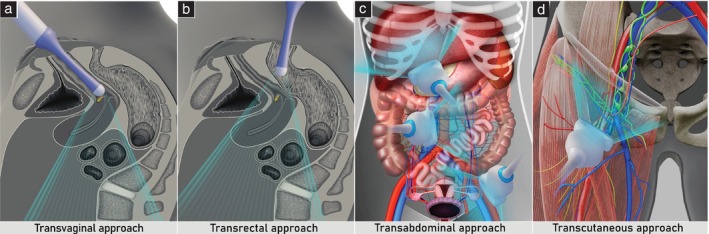
Ultrasound approaches for assessing infradiaphragmatic lymph nodes. Schematic diagrams showing transvaginal (a), transrectal (b), transabdominal (c) and transcutaneous (d) approaches. These are combined during ultrasound examination of gynecological cancers, allowing detailed assessment of the infradiaphragmatic lymph‐node regions.

The lymph nodes most frequently involved in gynecological cancer are the infradiaphragmatic lymph nodes, including the abdominopelvic parietal (retroperitoneal), visceral and inguinal lymph nodes. In patients referred for a specialized ultrasound examination for suspected gynecological cancer, these groups of lymph nodes should be a routine part of the initial assessment (Figure 3).

The ultrasound examination should commence with the use of an endocavitary transducer (≥ 5 MHz), which enables high‐resolution imaging of the pelvic structures, including the parietal and visceral lymph nodes. Endovaginal insertion of the endocavitary probe is preferable (transvaginal approach); however, in cases of vaginal stenosis (i.e. after radiation therapy), *virgo intacta* or cervical/vaginal cancer, an endoanal insertion is preferred (transrectal approach) (Figure 3). Transvaginal and transrectal sonography enable proximity of the probe to the pelvic lymph nodes without significant acoustic limitations. Next, a convex‐array transducer (up to 9 MHz) is recommended to complete the assessment of the pelvic lymph nodes and to assess the abdominal (parietal and visceral) lymph nodes using a transabdominal approach. In contrast to transvaginal or transrectal sonography, transabdominal sonography may sometimes be technically difficult due to conditions such as obesity, chronic ileus or tense ascites.

Finally, a high‐frequency linear‐array probe (7.5–15 MHz) is used, with a transcutaneous approach, for evaluation of the peripheral lymph nodes. These include the inguinal nodes, as well as any non‐peripheral lymph nodes, such as mesenteric lymph nodes, if they are in close proximity to the probe (Figure 3).

### Parietal (retroperitoneal) lymph nodes

The parietal (retroperitoneal) lymph nodes are located posterior to the peritoneum and along the major abdominal blood vessels. They are part of a group of nodes that spreads from the pelvis to the inferior surface of the diaphragm and can be divided into pelvic (iliac) and abdominal (lumbar) lymph nodes. The pelvic lymph nodes drain the pelvic organs, and they also collect lymphatic fluid from the inguinal and lower‐limb nodes^8^, ^9^. The abdominal lymph nodes drain primarily the retroperitoneal organs, such as the kidneys, adrenal glands and gonads, but also the intra‐abdominal organs, such as the liver and spleen, and they also receive lymphatic vessels from the pelvic lymph nodes^9^.

#### Anatomical classification of parietal lymph nodes

**Figure 4:**
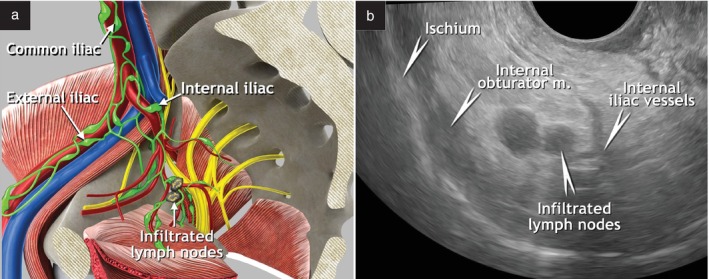
Pelvic parietal (iliac) lymph nodes. (a) Schematic diagram showing retroperitoneal pelvic lymph nodes: external iliac nodes, including obturator nodes; internal iliac nodes, including sacral nodes; and common iliac nodes. An example of infiltrated lymph nodes, along the course of the internal iliac vessels in proximity to the uterine artery, is shown. (b) Corresponding ultrasound findings of infiltrated lymph nodes along the course of the internal iliac vessels, demonstrated during transvaginal ultrasound imaging (transverse plane), using 4–9‐MHz endocavitary probe. This clinical case is presented in Videoclip S1. m., muscle.

The pelvic parietal (iliac) lymph nodes can be divided into common iliac, external iliac and internal iliac (hypogastric) nodes, according to their location along the iliac artery and vein (Figure 4)^9^. Following the recommended classification system found in the *Terminologia Anatomica* ^7^, obturator lymph nodes within the obturator fossa belong to the external iliac lymph nodes, and the sacral lymph nodes belong to the internal iliac lymph nodes^10^, ^11^.

**Figure 5:**
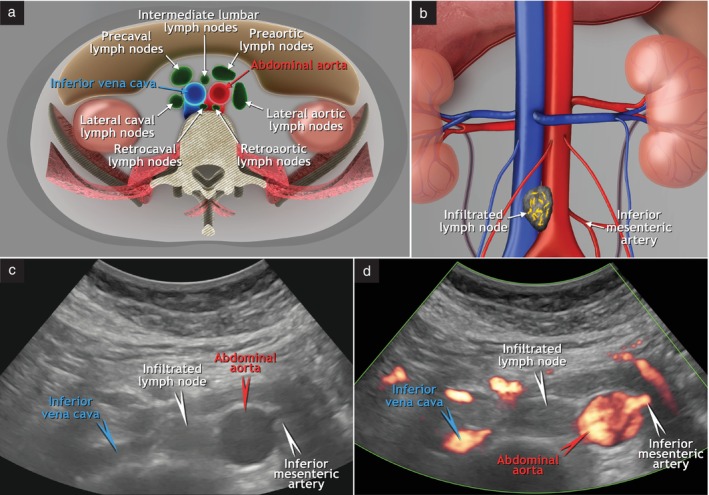
Abdominal parietal (lumbar) lymph nodes. (a) Schematic diagram showing the abdominal parietal (lumbar) lymph nodes, from left to right: lateral aortic, pre‐ and retroaortic, intermediate lumbar (interaortocaval), retro‐ and precaval and lateral caval lymph nodes. (b) Schematic diagram showing example of infiltrated intermediate lumbar lymph node. Note that the origin of the inferior mesenteric artery further divides abdominal lymph nodes into inframesenteric and supramesenteric lymph nodes. (c,d) Corresponding ultrasound findings from transabdominal ultrasound examination (transverse plane) using 2–9‐MHz convex‐array probe: grayscale (c) and color Doppler (d) imaging of infiltrated intermediate lumbar supramesenteric lymph node. This clinical case is presented in Videoclip S3.

The abdominal parietal (lumbar) lymph nodes can be divided into the lateral aortic (to the left of the abdominal aorta), preaortic (ventral to the aorta), post/retroaortic (dorsal to the aorta), intermediate lumbar (interaortocaval, between the aorta and the inferior vena cava), precaval (ventral to the inferior vena cava), post/retrocaval (dorsal to the inferior vena cava) and lateral caval (to the right of the inferior vena cava) lymph nodes (Figure 5)^9^. They can be further classified into inframesenteric and supramesenteric in relation to the origin of the inferior mesenteric artery^11^. The abdominal lymph nodes also include the renal hilar and retrocrural lymph nodes, although the latter may sometimes be difficult to assess on ultrasound due to their deep location and overlying organs, as they are located behind the crura at the level of the aortic hiatus where the abdominal aorta becomes the thoracic aorta. Abdominal lymph nodes drain into the lumbar lymph trunks, which ultimately lead to the cisterna chyli and the thoracic duct^9^. It is crucial to emphasize that, according to common surgical terminology, the lumbar lymph nodes surrounding the aorta and inferior vena cava are frequently called ‘para‐aortic’ lymph nodes. However, according to the *Terminologia Anatomica* ^7^, the para‐aortic lymph nodes correspond only to lateral aortic lymph nodes. To avoid ambiguity, we recommend adhering to the latter nomenclature, adopting precise and standardized terminology^7^.

#### Examination technique

Ultrasound assessment of lymph nodes in the abdominopelvic cavity should start with a pelvic ultrasound examination. Pelvic parietal lymph nodes can be visualized effectively in high resolution using either a transvaginal or a transrectal approach. A transabdominal approach using a convex‐array transducer should also be used, to ensure that no nodes located ventrally on the external iliac vessels or close to the lacuna vasorum (i.e. out of the field‐of‐view of the endocavitary probe) are missed.

**Figure 6:**
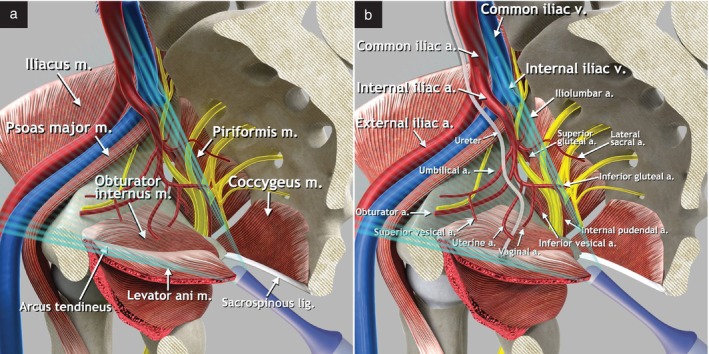
Schematic diagrams showing relevant structures of the pelvic sidewall assessable during transvaginal/transrectal ultrasound imaging. Pelvic muscles (a) and arteries (b) are shown. a., artery; lig., ligament; m., muscle; v., vein.

The ultrasound examination starts with the use of an endocavitary transducer, with the patient lying in a dorsal lithotomy position and the bladder ideally emptied prior to assessment (Figure 3). Using the systematic assessment of the pelvic sidewall described recently by Fischerova *et al*.^12^, it is possible to perform a systematic evaluation of the pelvic sidewall vessels and detect parietal lymph nodes, when apparent due to their reaction to a pathological insult (e.g. inflammation, prior invasive procedure, malignancy), located along the branches of the external and internal iliac vessels (Figure 6; for a further detailed explanation of the examination technique, please refer to videoclip S3 in Fischerova *et al*.^12^). The first step is to identify the uterine vessels lateral to the cervix. The second step is to detect the branches of the anterior division of the internal iliac artery, including the obturator artery, adjacent to which it is often possible to find pathological lymph nodes in several gynecological tumors^8^. The third step is to identify the branches of the posterior division of the internal iliac artery. The fourth and final step is to follow the anterior and posterior branches until the interiliac bifurcation between the internal and external iliac arteries. From that point, it is important to visualize the complete course of the external iliac artery up to the vascular lacuna, which is the medial compartment beneath the inguinal ligament allowing the passage of major vessels and lymphatics between the inguinal region and the pelvis, to ensure that no external iliac node near the vascular lacuna of the inguinal canal is missed. The node affected most consistently is located within the femoral ring (medial lacunar node or Cloquet's or Rosenmüller's node). Along with the arteries, the corresponding veins are observed. During the scan, the dynamic nature of the examination can also be used to determine if the lymph node appears fixed to the adjacent vessel wall or other structures; this could be valuable in predicting vascular or other pelvic structural involvement. Examples of abnormal ultrasound findings related to the pelvic parietal lymph nodes are shown in Videoclip S1.

**Figure 7:**
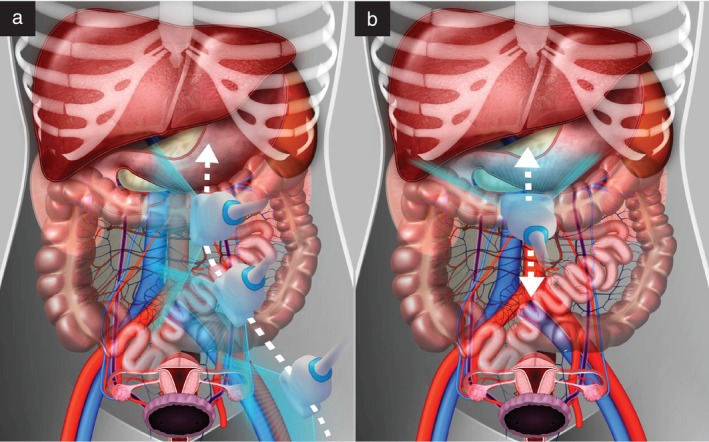
Schematic diagrams showing ultrasound methodology for evaluation of pelvic and abdominal parietal lymph nodes by transabdominal scan. White dotted line shows the direction of movement of the probe during the scan. Examination of pelvic lymph nodes in the oblique plane (a), and abdominal lymph nodes in the longitudinal plane (a) in combination with the transverse plane (b).

Next, a convex‐array transducer is recommended to complete the assessment of the pelvic and abdominal parietal lymph nodes (Figure 7). The transducer is placed on the abdominal wall with the patient lying in a dorsal lithotomy position (Videoclip S2). The examination starts at the inguinal ligament to identify the femoral vessels running beneath it to become the external iliac vessels^11^. The transducer is oriented in an oblique plane and moved along the large iliac vessels up to the aortic bifurcation. The external iliac artery is visualized ventral to the external iliac vein on the psoas major muscle. The vein is identified due to its collapsibility upon applying gentle pressure with the transducer. During the examination of the iliac lymph nodes in the oblique plane, the probe should be swept slightly both medially and laterally over the vessels, to ensure that no lymph nodes are missed^11^. It is also essential to examine the obturator lymph nodes within the obturator fossa, which lies dorsal to the external iliac vessels. The bifurcation of the internal and external iliac vessels is at the pelvic brim and the ureter may also be visualized here, crossing the iliac vessels towards the pelvis. Following the common iliac vessels along the medial border of the psoas major muscle, the probe should be moved cranially, passing the bifurcations of the inferior vena cava and aorta at the level of the fifth and fourth lumbar vertebra, respectively^11^. From the aortic bifurcation up to the diaphragm, the transducer is first oriented in the longitudinal plane to scan the lymph nodes along the abdominal aorta and inferior vena cava^11^. As described for the pelvis, the transducer should be swept slightly and tilted medially and laterally to include the lymph‐node regions along the sides of the inferior vena cava and abdominal aorta^11^. Next, the lumbar lymph nodes are evaluated in the transverse plane. Each visible lymph node should be identified in both longitudinal and transverse planes, measured and described using VITA terminology (Figures 7 and S1, Table S1). Examples of abnormal ultrasound findings related to the abdominal parietal lymph nodes are shown in Videoclip S3.

### Visceral lymph nodes

The pelvic visceral lymph nodes are located around their corresponding viscera^9^. The afferent lymph vessels arise from their respective organs and run along the vascular channels into the visceral lymph nodes. Their efferent lymph vessels drain from the visceral lymph nodes into the internal and external iliac lymph‐node groups. The final ascending pathways lead into the abdominal (lumbar) lymph nodes^9^. The abdominal visceral lymph nodes are located around the origin and branches of the celiac trunk and superior mesenteric and inferior mesenteric arteries^10^. Their efferent lymph vessels contribute to the intestinal lymph trunk and drain into the cisterna chyli and thoracic duct^10^.

#### Anatomical classification of visceral lymph nodes

**Figure 8:**
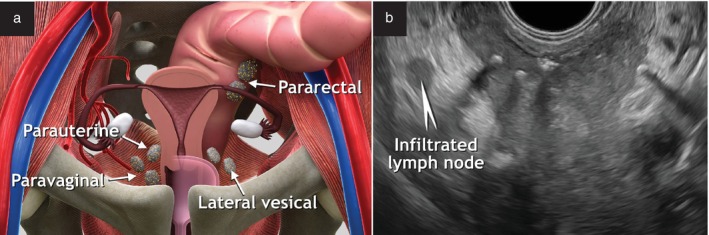
Pelvic visceral lymph nodes. (a) Schematic diagram showing examples of pelvic visceral lymph nodes, including pararectal, parauterine, paravaginal and lateral vesical nodes. (b) Ultrasound findings (transverse plane) of parauterine infiltrated lymph node in the right paracervix, using a transrectal approach with a 4–9‐MHz endocavitary probe.

The pelvic visceral lymph nodes are classified with reference to their respective internal organs (Figure 8)^9^. Lymph nodes in the subperitoneal tissue surrounding the bladder are described as lateral vesical (lateral to the bladder), prevesical (ventral to the bladder) and postvesical (dorsal to the bladder) groups. The lymph nodes adjacent to the vagina, cervix and uterine body are described as the paravaginal and parauterine nodes. The posterior pelvic compartment includes the pararectal lymph nodes, located around the middle rectal vessels on both sides of the rectum, and the visceral lymph nodes along the sigmoid colon (sigmoid nodes), following the course of the sigmoid vessels^9^. There is no definite anatomical landmark to distinguish parietal from visceral lymph nodes located alongside the internal iliac vessels; therefore, both should be regarded as regional lymph nodes.

**Figure 9:**
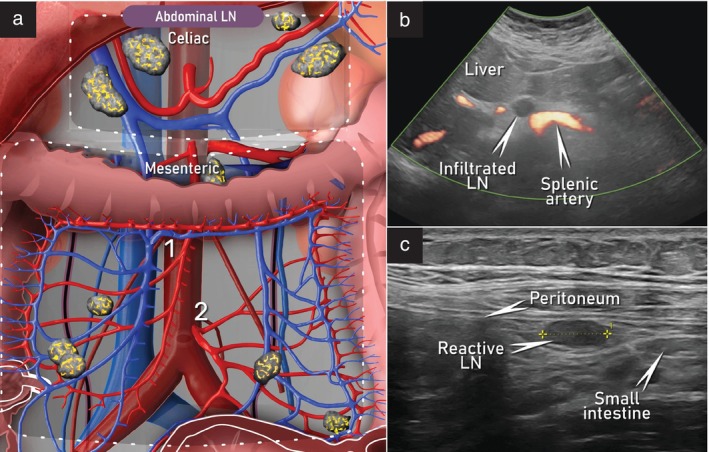
Abdominal visceral lymph nodes. (a) Schematic diagram showing abdominal visceral nodes, including celiac and superior (1) and inferior (2) mesenteric nodes. (b) Appearance on ultrasound imaging of infiltrated celiac lymph node, demonstrated by transabdominal scan (transverse plane) using 2–9‐MHz convex‐array probe. (c) Appearance on ultrasound imaging of reactive mesenteric lymph node (calipers) (transverse plane), using high‐resolution 4.5–15‐MHz linear‐array probe, placed on the anterior wall of the right lumbar region. This approach adds a detailed view of nodal architecture in situations in which the node is located superficially and is in close proximity to the probe. LN, lymph node(s).

The abdominal visceral lymph nodes form a chain divided into three groups based on their proximity to the celiac, superior mesenteric and inferior mesenteric vessels (Figure 9)^11^. The lymph nodes located ventral to the aorta around the origin of the celiac trunk from the aorta are described as the preaortic parietal lymph nodes, while those alongside the branches of the celiac trunk up to the splenic vessels and the hepatic hilum are termed the visceral celiac lymph nodes. Branches of the celiac trunk are the left gastric, common hepatic and splenic arteries. The celiac lymph nodes receive lymphatic channels from the stomach, majority of the duodenum (D1, D2, D3), major part of the liver, gallbladder, pancreas and spleen. They also receive lymphatic vessels from the superior and inferior mesenteric nodes^10^. The superior mesenteric nodes drain the terminal part of the duodenum (D4), head, neck and uncinate process of the pancreas, the small intestine and the right colon^10^. The inferior mesenteric nodes receive the lymphatic vessels from the upper part of the rectum and left colon^10^. The involvement of mesenteric and celiac lymph nodes in gynecological tumors, particularly in high‐grade serous tubo‐ovarian carcinoma, is associated primarily with lymphatic drainage from the preaortic, precaval, interaortocaval and other lumbar lymph nodes (which are involved primarily as a result of cancer spread via the infundibulopelvic pathway). A direct association has also been demonstrated between intestinal wall infiltration and the risk of mesenteric lymph‐node involvement^13^.

#### Examination technique

The visceral lymph nodes are evaluated alongside the course of the visceral vessels draining the visceral organs^14^. The pelvic visceral lymph nodes are assessed with an endocavitary transducer during transvaginal/transrectal pelvic ultrasound examination and are evaluated together with the pelvic parietal lymph nodes. The probe is placed in the anterior fornix of the vagina. From the midsagittal plane at the level of the internal cervical os, the probe is rotated 90° counterclockwise to obtain the transverse plane and to identify the uterine vessels. The parauterine lymph nodes alongside the uterine vessels can thus be detected and, by slight withdrawal of the probe, the paravaginal lymph nodes alongside the vaginal vessels can be visualized. The methodology of ultrasound assessment of visceral branches of the internal iliac vessels has been described recently by our group; for a further detailed explanation of the examination technique, please refer to videoclip S3 in Fischerova *et al*.^12^. Examples of abnormal ultrasound findings related to the pelvic visceral lymph nodes are shown in Videoclip S4.

The abdominal visceral lymph nodes are evaluated using a transabdominal approach with a convex‐array transducer and, if acoustic conditions permit, also with a linear‐array probe, with the patient lying in the supine position (Figure 9). Evaluation of celiac lymph nodes is a routine part of upper abdominal scanning, during which the course of the celiac trunk is followed from its origin up to the hepatic and splenic hila^14^. Assessment of the mesenteric lymph nodes is carried out after the assessment of the abdominal parietal lymph nodes, as presented in Videoclip S2. The probe is positioned in the longitudinal plane at the level of the origin of the superior mesenteric artery, approximately 1 cm caudal to the celiac trunk and ventral to the aorta, and then swept caudally to follow the course of the superior mesenteric artery^14^. Any abnormal lymph node observed must also be confirmed and assessed in the transverse plane. The superior mesenteric artery runs posterior to and under the neck of the pancreas. Both superior and inferior mesenteric arteries descend ventral to the aorta and deviate laterally, giving rise to their branches. The superior mesenteric artery runs along the root of the mesentery, downwards to the right, supplying the bowel from the mid descending duodenum to the distal third of the transverse colon^11^. The superior mesenteric vein lies to the right of the superior mesenteric artery. The inferior mesenteric artery runs within the mesentery of the hindgut, downwards to the left, where it branches. It supplies the distal transverse colon up to the proximal anal canal^11^. The inferior mesenteric vein runs alongside the corresponding artery, draining into the splenic vein. The confluence of the splenic vein with the superior mesenteric vein forms the portal vein. Following the main courses of the mesenteric vessels, both sides of the mesentery of the small intestine can be evaluated. Examples of abnormal ultrasound findings related to the abdominal visceral lymph nodes are shown in Videoclip S5.

### Inguinal lymph nodes

The inguinal lymph nodes receive afferent lymphatics from the gluteal region, anterior abdominal wall, external genitals (including the distal third of the vagina), anal canal, perianal region and lower limbs^11^.

#### Anatomical classification of inguinal lymph nodes

**Figure 10:**
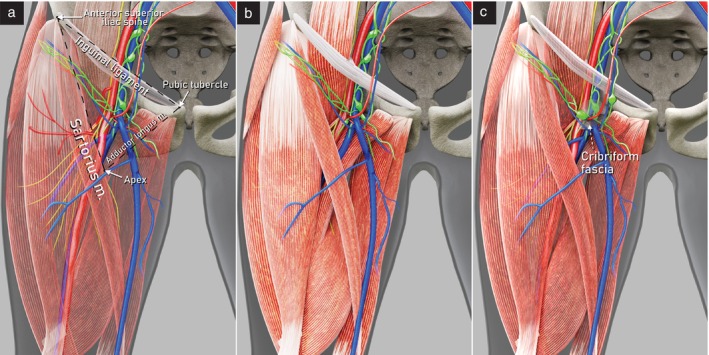
Inguinal lymph nodes. Schematic diagrams showing: (a) anatomical landmarks of femoral triangle; (b) deep inguinal lymph nodes lying underneath the cribriform fascia (cribriform fascia has been omitted from this image); and (c) superficial inguinal lymph nodes lying along the great saphenous vein and its tributaries, superficial to the cribriform fascia. m., muscle.

Inguinal lymph nodes are distributed within the femoral triangle (Scarpa's triangle). The base of the triangle is the inguinal ligament, the lateral side is the medial border of the sartorius muscle, and the medial side is the lateral border of the adductor longus muscle (Figure 10). The floor consists of the iliopsoas muscle laterally and the pectineus muscle medially. The apex of the triangle corresponds caudally to the intersection of the sartorius and long adductor muscles^5^, ^11^.

**Figure 11:**
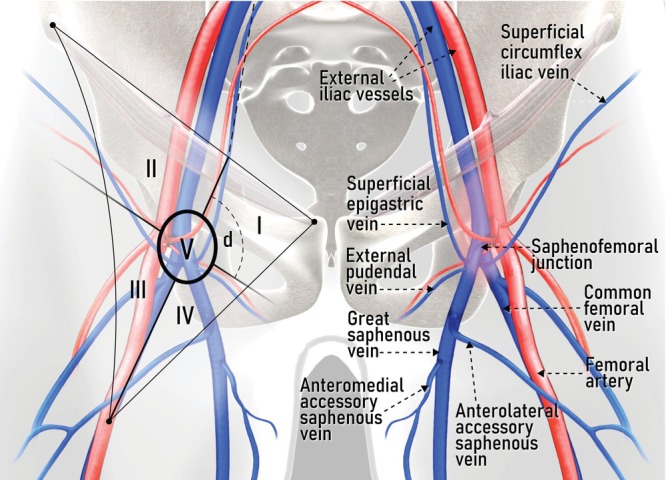
Schematic diagram showing Daseler regions^15^ I to V (right thigh), and relevant pelvic and inguinal vessels (left thigh). d, deep inguinal lymph nodes.

Inguinal lymph nodes are divided into superficial and deep groups. The superficial lymph nodes are found in the fatty tissue between the superficial fascia (also called Camper's fascia), which is the deep boundary of the hypodermal layer, and the deep fascia (also called fascia lata), which covers the ventral surface of the muscles. They are distributed along the saphenous vein and its tributaries (i.e. superficial inferior epigastric, superficial circumflex iliac, superficial external pudendal and accessory anterolateral and anteromedial veins). The deep lymph nodes are located exclusively within the fossa ovalis, under the cribriform fascia, cranial to the saphenofemoral junction and medial to the femoral vein. Furthermore, to better describe the location of the inguinal lymph nodes, the femoral triangle can be subdivided into five Daseler regions by drawing a virtual line along the femoral vein and a second virtual perpendicular line at the level of the saphenofemoral junction, with, additionally, a circle around the center of the cross (Figure 11): superomedial region (I); superolateral region (II); inferolateral region (III); inferomedial region (IV); and central region (V)^15^.

#### Examination technique

**Figure 12:**
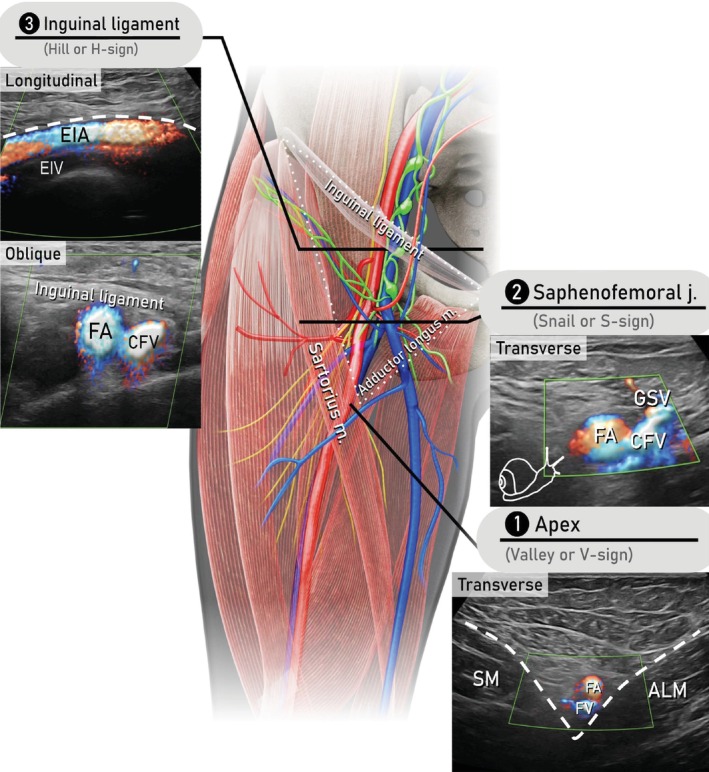
Schematic diagram showing anatomy of the femoral triangle (bordered by dotted lines, right thigh) and images showing corresponding ultrasound findings, using a transcutaneous approach with a linear‐array probe (7.5–15 MHz). Useful landmarks to assess the lymph nodes located in the femoral triangle are indicated. The scan starts from the apex (the lowest point) of the triangle, crosses the saphenofemoral junction and finally reaches the inguinal ligament. The vascular lacuna is represented by the medial compartment beneath the inguinal ligament containing the femoral vessels, lymph vessels and lymph nodes. Note, in the schematic diagram, the black horizontal lines define the level at which the probe is placed and not its orientation. The dashed lines highlight the shapes of the signs. ALM, adductor longus muscle; CFV, common femoral vein; EIA, external iliac artery; EIV, external iliac vein; FA, femoral artery; FV, femoral vein; GSV, great saphenous vein; j., junction; m., muscle; SM, sartorius muscle. (See also Videoclip S6.)

Before beginning the scan, the skin projection of the boundaries of the femoral triangle should be identified virtually, as demonstrated in Videoclip S6. The three vertices of the triangle are the anterior superior iliac spine, the pubic tubercle and the apex caudally. The apex is palpable as a softer point on the caudal and medial border of the upper third of the thigh (Figure 10a). It is recommended to use a high‐frequency linear‐array transducer. The patient lies supine, with legs extended, abducted and extrarotated. The examination starts with the probe in a transverse position at the apex of the femoral triangle (Figure 12). The first landmark is the ‘valley sign’ (V‐sign), which is formed by the medial and lateral sides of the triangle. Femoral vessels lie on the floor of the ‘V’, as in the bed of a canyon, and can also be identified using color or power Doppler. Moving the probe upwards in the transverse plane, Daseler regions III and IV are examined, separated in the middle by the cross‐section of the femoral vessels. Reaching the center of the triangle at the saphenofemoral junction, the second landmark is visualized, the ‘snail sign’ (S‐sign). The snail is composed of the transverse section of the following vessels: the last segment of the great saphenous vein (head), the tributary veins that enter the great saphenous vein (horns), the common femoral vein (body and tail) and the femoral artery (shell). This landmark represents the central reference point from which to explore all Daseler regions. The cranial limit of the examination is the vascular lacuna, below the inguinal ligament, where the femoral vessels continue into the pelvis, becoming the external iliac vessels. The inguinal ligament can be identified as a thick, hyperechogenic band that appears in an oblique scan, when the probe is aligned along a virtual line connecting the anterior superior iliac spine with the pubic tubercle. Here, rotating the probe along the vessels in a longitudinal scan the third landmark, the ‘hill sign’ (H‐sign) is observed. The vessels are seen curving gently, following the curvature of the upper branch of the superior pubic ramus, towards the bottom of the pelvis, as if down the slope of a hill. The top of the hill defines the pelvic inlet.

**Figure 13:**
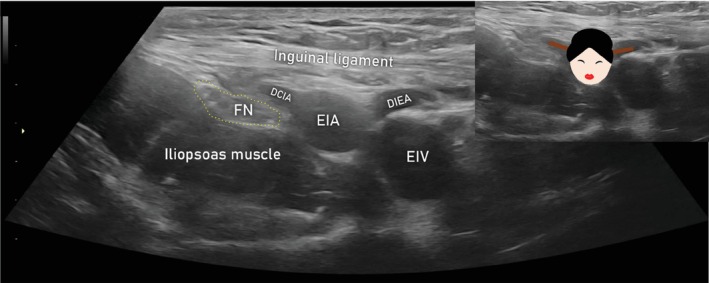
Geisha sign. Images illustrating ultrasound appearance of this novel sign, obtained using a linear‐array probe (7.5–15 MHz, transcutaneous approach). This sign marks the limits between the femoral and external iliac arteries on the right. DCIA, deep circumflex iliac artery; DIEA, deep inferior epigastric artery; EIA, external iliac artery; EIV, external iliac vein; FN, femoral nerve. (See also Videoclip S6.)

Another sign that can be used to identify the transition between femoral and external iliac vessels is the ‘Geisha sign’ (Figure 13). In the transverse section, the external iliac artery (Geisha's head) is observed to give rise to two coaxial branches, the deep circumflex iliac artery laterally and the deep inferior epigastric artery medially (Geisha's rods, called kanzashi). The corresponding veins run parallel to the arteries.

**Figure 14:**
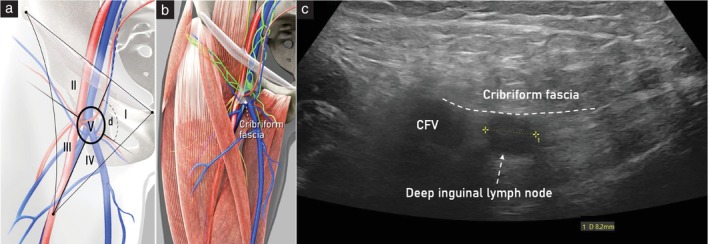
Deep inguinal lymph nodes (right thigh). (a) Schematic diagram showing Daseler regions I to V; the deep inguinal lymph nodes (d) are located in Daseler region I. (b) Schematic diagram showing the location of these nodes underneath the cribriform fascia. (c) Ultrasound findings of a suspicious deep inguinal lymph node (calipers), exhibiting round shape and absence of nodal‐core sign, obtained using a linear‐array probe (7.5–15 MHz) and a transcutaneous approach. The cribriform fascia (dashed line) is not easily detectable with ultrasound. It runs at the caudal margin of the saphenofemoral junction, parallel to the superficial fascia. CFV, common femoral vein.

Finally, superficial lymph nodes are found within all five Daseler regions, between Camper's fascia, which is a hyperechogenic line that runs parallel to the skin, and the fascia lata, a hyperechogenic line that covers the ventral surface of the muscles. Conversely, to locate the deep inguinal lymph nodes within the oval fossa (Daseler region I), a line parallel to the femoral fascia should be followed, precisely at the level of the saphenofemoral junction, since the cribriform fascia is not visible on ultrasound (Figure 14). Examples of abnormal ultrasound findings related to the inguinal lymph nodes are shown in Videoclip S7.

## SUPRADIAPHRAGMATIC LYMPH NODES

Assessment of supradiaphragmatic lymph nodes (the superior diaphragmatic (cardiophrenic), supraclavicular, axillary and parasternal (internal thoracic/mammary)) lymph nodes is not routine, but should be performed in the presence of risk factors predisposing for their infiltration, or when there are relevant symptoms or palpable nodes^16^. This group of lymph nodes is not commonly involved in gynecological cancer but should be examined whenever there is risk of distant lymphatic spread, such as in the presence of extensive inguinal and/or abdominal lymphadenopathy or subdiaphragmatic or pleural involvement due to carcinomatosis^16^, ^17^.

**Figure 15:**
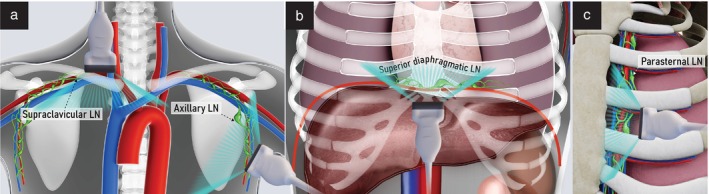
Supradiaphragmatic lymph nodes. Schematic diagrams showing placement of the linear‐array probe for ultrasound evaluation of supradiaphragmatic lymph nodes (transcutaneous approach). This is the preferred probe for assessment of supraclavicular, axillary and parasternal lymph nodes. For superior diaphragmatic lymph nodes, either a convex‐array probe or a linear‐array probe can be used. (a) Supraclavicular and axillary lymph nodes. (b) Superior diaphragmatic lymph nodes. (c) Parasternal lymph nodes. LN, lymph node(s).

To assess the superior diaphragmatic (cardiophrenic) lymph nodes, a convex‐array transducer (up to 9 MHz) or linear‐array probe is used, depending on the acoustic conditions. For lymph nodes in the supraclavicular, axillary and parasternal region, a high‐frequency linear‐array transducer (7.5–15 MHz), as for inguinal lymph nodes, should be used (Figure 15).

### Superior diaphragmatic (cardiophrenic) lymph nodes

Superior diaphragmatic (cardiophrenic) lymph nodes represent the caudal portion of the mediastinal lymph nodes and are located within the fatty tissue surrounding the base of the heart in an extrapleural space^18^. Superior diaphragmatic lymph nodes can be divided into three groups: anterior, lateral and posterior. The drainage pathway differs between the groups. The anterior group drains the anterior chest, supra‐umbilical abdominal wall, anterior diaphragm, liver surface and medial portion of the breasts; the lymph drained from the anterior group passes through the prepericardial and parasternal lymph nodes. The lateral group is responsible for draining lymph from the intrathoracic organs. The posterior group collects lymph from the chest wall, posterior pleura, esophagus and posterior diaphragm^18^. The lymphatic vessel connections between the subperitoneal infradiaphragmatic and the supradiaphragmatic lymph nodes are responsible for the occurrence of cardiophrenic metastases if diaphragmatic carcinomatosis is present^17^, ^19^. The superior diaphragmatic efferent lymph vessels drain into the right and left anterior mediastinal chains, which further drain into the right lymphatic duct and the thoracic duct on the left, and into the paramammary and parasternal (internal mammary) nodes.

It is essential to highlight that the terminology used in the literature for these lymph nodes has often been variable and misleading. For example, ‘cardiophrenic’ is the most commonly used term in surgical terminology, yet this group of parietal thoracic lymph nodes has also often been referred to as ‘anterior diaphragmatic’, ‘supradiaphragmatic’, ‘prepericardial’, ‘pericardial’ or ‘retrosternal’^20^, ^21^. According to anatomical terminology, the correct term for describing these lymph nodes is ‘superior diaphragmatic lymph nodes’, and this, therefore, is the term used herein^7^.

#### Anatomical classification of superior diaphragmatic lymph nodes

**Figure 16:**
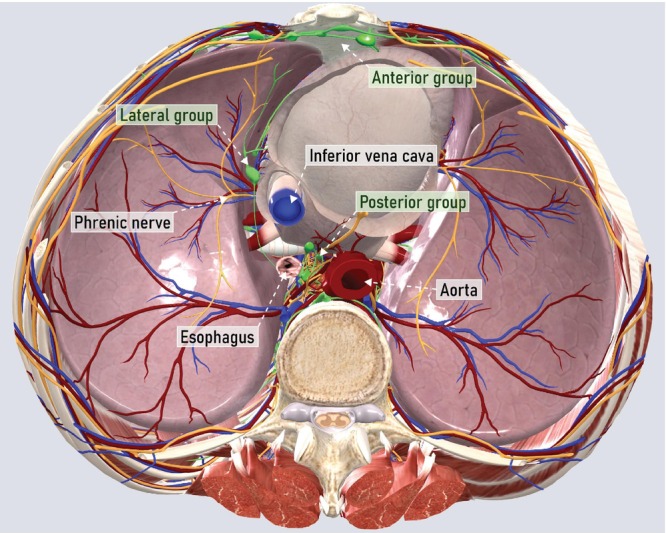
Superior diaphragmatic (cardiophrenic) lymph nodes (inferior (abdominal) view). Schematic diagram (transverse plane, the diaphragm is removed) showing the three groups of superior diaphragmatic lymph nodes in axial view: the anterior group is located between the xiphoid process of the sternum and the costochondral articulation of the 7^th^ rib and the pericardium; the lateral group is between the pericardium medially and the lung hilum laterally; and the posterior group is next to the esophagus.

Superior diaphragmatic (cardiophrenic) lymph nodes can be found in the fatty tissue of the lower part of the mediastinum, bordered by the base of the heart, the diaphragm and the chest wall (Figure 16)^22^. These are classified into three groups, based on their location in relation to the heart. The anterior group (anterior diaphragmatic) comprises a few lymph nodes located between the xiphoid process of the sternum and the costochondral articulation of the seventh rib and the pericardium. The middle or lateral group (lateropericardial) is located between the pericardium medially and the lung hilum laterally, where the phrenic nerves run. The posterior (juxtaesophageal) group can be found next to the esophagus, posteromedial to the inferior vena cava^18^. Metastatic involvement of the superior diaphragmatic space may occur in advanced tubo‐ovarian carcinoma with extensive diaphragmatic carcinomatosis and mostly affects the anterior compartment^22^. The posterior group could also be involved in cases of abdominopelvic cancer due to the connection with retrocrural and lumbar (especially lateral aortic) lymph nodes, although infiltration of this group is relatively uncommon^23^.

#### Examination technique

**Figure 17:**
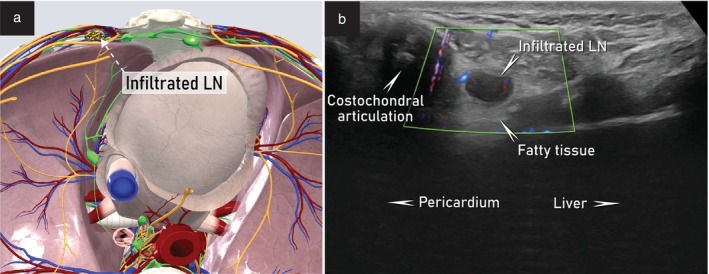
Anterior diaphragmatic lymph nodes. (a) Schematic diagram showing example of infiltrated node of the anterior group of superior diaphragmatic lymph nodes. (b) Corresponding ultrasound appearance of infiltrated superior diaphragmatic lymph node, assessed using transcutaneous approach with a 4.5–15‐MHz linear‐array probe. This clinical case is presented in Videoclip S9. LN, lymph node.

To assess the superior diaphragmatic lymph nodes, the patient lies in a supine position (Videoclip S8). Either the linear‐array or the convex‐array transducer can be used, according to patient suitability. The probe is first positioned in the transverse plane and then tilted to follow the costal arch on both sides in an oblique view. The region of interest is located between the xiphoid process of the sternum, the costochondral articulation of the seventh rib and the pericardium. The probe is then rotated to obtain the longitudinal plane under the xiphoid process of the sternum for a subxiphoid view. The space between the xiphoid process, the pericardium and the diaphragm is examined in the longitudinal plane and the anterior lymph‐node group is assessed, moving the probe laterally and medially (Figure 17). For ultrasound assessment of the posterior group of superior diaphragmatic lymph nodes, the probe is moved towards the midline in a longitudinal plane. The high frequency of the linear‐array probe limits the depth of penetration for evaluation of this group of lymph nodes, so a convex‐array probe is preferable. The gastroesophageal junction must be identified by tilting the probe in an oblique view. Affected lymph nodes may be found in the region next to the esophagus, posteromedial to the inferior vena cava, above the diaphragm. However, this region may be difficult to visualize with ultrasound due to the inherently poor acoustic conditions; therefore, if there is any suspicion of involvement, an alternative imaging modality, such as computed tomographic scan of the thorax, should be performed. Ultrasound assessment of the lateral group of superior diaphragmatic lymph nodes is not performed routinely due to their location between the lung hilum and the lateral side of the heart. Examples of abnormal ultrasound findings related to the superior diaphragmatic lymph nodes are shown in Videoclip S9.

### Supraclavicular lymph nodes

The majority of the body's lymphatic fluid is collected via the thoracic duct, which receives lymph from the abdominopelvic organs, left hemithorax, left arm and left part of the head and neck, before entering the venous angle between the left subclavian and internal jugular veins. Involvement of the left supraclavicular lymph nodes, known eponymously as ‘Virchow's node(s)’, may be the first visible sign of ‘occult’ malignant spread (Troisier sign) from various gynecological cancers^24^. Virchow's node is the most proximal node (the end node) of the left supraclavicular group and is located near the venous angle, lying ventral to the anterior scalene muscle and dorsal to the sternocleidomastoid and platysma muscles^25^. On the right side of the body, the right lymphatic duct receives lymph from the diaphragmatic surface of the liver, right hemithorax, right arm, and right part of the head and neck. Metastatic involvement of the right supraclavicular nodes by gynecological malignancy is uncommon^26^. Another possible pathway of infiltration of the supraclavicular lymph nodes from gynecological cancer is via the lymph vessels lying along the inferior epigastric vessels. These vessels anastomose with the superior epigastric vessels, which are the terminal branches of the internal thoracic vessels. Lymph vessels that follow the internal thoracic vessels drain into the parasternal (internal mammary) nodes and finally into the supraclavicular nodes. The lateral axillary nodes may also drain into the supraclavicular nodes.

#### Anatomical classification of supraclavicular lymph nodes

**Figure 18:**
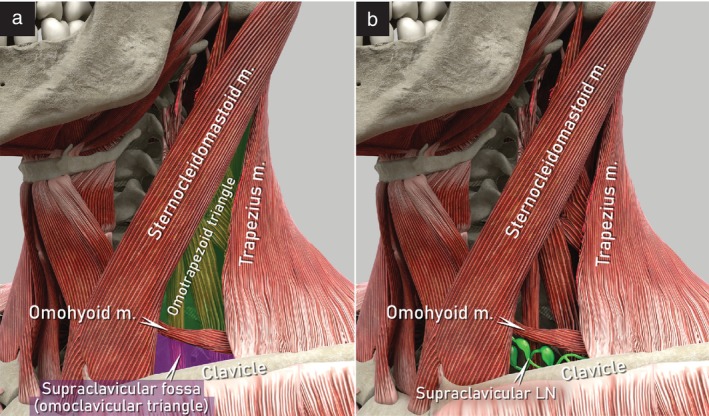
Supraclavicular lymph nodes: schematic diagrams showing lateral cervical region. (a) Supraclavicular fossa (purple, also known as omoclavicular triangle) and omotrapezoid triangle (green). (b) Supraclavicular (also known as scalene) lymph nodes. LN, lymph node(s); m., muscle.

The supraclavicular (scalene) lymph nodes, including Virchow's node, are included in the group of lymph nodes in the lateral neck region^27^. The supraclavicular nodes can be found in the supraclavicular fossa (omoclavicular triangle), located under the level of the cricoid cartilage, delimited by the omohyoid muscle cranially, the superior border of the clavicle caudally, the medial (anterior) border of the trapezius muscle laterally and the lateral (posterior) border of the sternocleidomastoid muscle medially (Figure 18)^28^, near the posterolateral part of the internal jugular vein.

#### Examination technique

For ultrasonographic examination of the neck, the patient lies in a supine position with the neck hyperextended and turned to the opposite side of the region being examined. A high‐frequency linear‐array transducer is used (Videoclip S10).

To assess the supraclavicular nodes, the supraclavicular fossa should be scanned in both transverse and longitudinal views. The probe is first placed in a transverse view at the caudal limit of the supraclavicular fossa, on the superior margin of the clavicle. The probe is then moved cranially along the sternocleidomastoid muscle, which forms the medial limit of the fossa. The lateral lobe of the thyroid gland is identified along with the omohyoid muscle, which inserts at the hyoid bone and originates from the superior border of the scapula. The omohyoid muscle is followed laterally, dorsal to the sternocleidomastoid muscle, delineating the cranial limit of the supraclavicular fossa, located under the level of the cricoid cartilage. The lateral limit is formed by the medial edge of the trapezius muscle, which can be identified by moving the probe further laterally.

**Figure 19:**
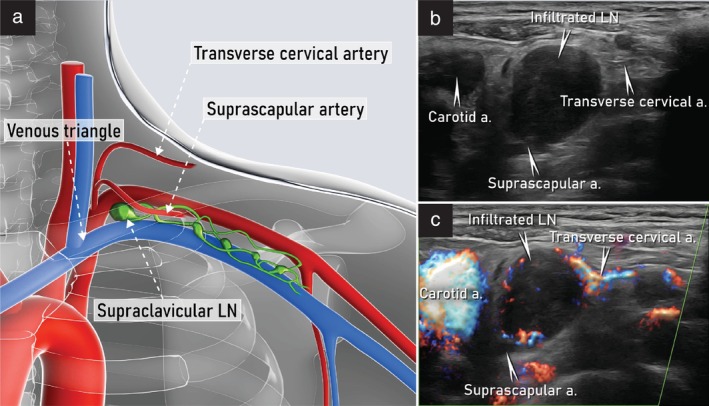
Supraclavicular lymph nodes. (a) Schematic diagram showing supraclavicular nodes and main vessels of lateral cervical region crossing supraclavicular fossa. Venous triangle is formed by convergence of subclavian and jugular veins. (b,c) Ultrasound appearance of infiltrated scalene lymph node on grayscale (b) and power Doppler (c) imaging. Note the presence of transcapsular flow in the infiltrated lymph node in (c). The internal jugular vein is not identifiable in this scan due to compression by the adjacent infiltrated lymph node. A transcutaneous approach with a linear‐array probe (7.5–15 MHz) placed in a transverse plane was used for both scans. a., artery; LN, lymph node(s).

The probe is then repositioned to obtain a longitudinal view of the supraclavicular fossa, starting from the common carotid artery and internal jugular vein and shifting laterally in a ‘fan‐shaped’ movement, to ensure complete assessment and better detection of any pathological findings (Figure 19). The lymph nodes can be found posterolateral to the internal jugular vein. Examples of abnormal ultrasound findings related to the supraclavicular lymph nodes are shown in Videoclip S11.

### Axillary lymph nodes

Axillary lymph nodes drain the breasts, thoracic wall, arms and subcutaneous tissues above the umbilicus^27^. Involvement of axillary lymph nodes in gynecological cancer is rare, and seen mostly in advanced or recurrent tubo‐ovarian carcinoma. Another lymphatic dissemination pathway that can lead to axillary nodal infiltration is the connection between infiltrated superficial inguinal lymph nodes and the lymphatics that run along the superficial inferior epigastric vessels towards the thoraco‐epigastric vein, reaching the ipsilateral axilla via the lateral thoracic vein^17^, ^29^, ^30^.

#### Anatomical classification of axillary lymph nodes

**Figure 20:**
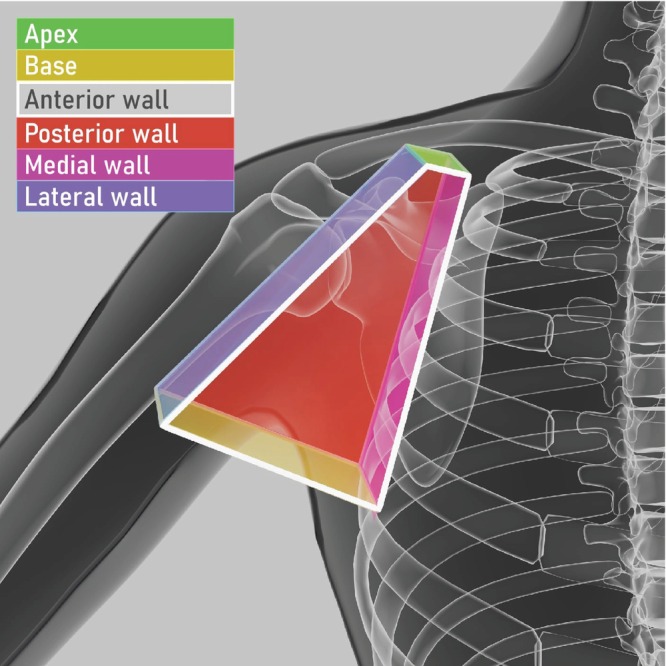
Axillary borders. Schematic diagram showing apex, lateral wall, medial wall, posterior wall, anterior wall and base. The apex is defined by the first rib, clavicle and scapula. The lateral wall is formed by the humerus, coracobrachialis muscle and short head of the biceps muscle. The medial wall is formed by the serratus anterior muscle and intercostal muscles. The posterior wall is formed mainly by the scapula, subscapularis muscle and, in the lower part, the teres major and latissimus dorsi muscles, which define the posterior axillary fold. The anterior wall is formed by the pectoralis major and pectoralis minor muscles, whose lateral edges define the anterior axillary fold. The base of the axilla is formed by the skin, adipose tissue and axillary fascia. (See also Videoclip S12.)

The axilla is shaped like a truncated trapezoid‐based pyramid, with an upper medial part (apex), a lower lateral part (base) and four sides (walls) (Figure 20, Videoclip S12). The truncated apex is also called the cervicoaxillary canal. It is bordered by the first rib, the clavicle and the scapula. The base is formed by the skin, adipose tissue and axillary fascia. The anterior wall is formed by the pectoralis major and pectoralis minor muscles, whose lateral edges define the anterior axillary fold. The posterior wall is formed mainly by the scapula, subscapularis muscle and, in the lower part, by the teres major and the latissimus dorsi muscles, which define the posterior axillary fold. The medial wall is formed by the serratus anterior muscle, which covers the upper four ribs and intercostal muscles. The lateral wall is formed by the humerus, the coracobrachialis muscle and the short head of the biceps muscle.

The axillary pyramid is crossed by axillary vessels and lymphatics. The axillary artery originates from the subclavian artery at the level of the first rib and becomes the brachial artery at the humeral insertion of the teres major muscle. It can be subdivided into three parts. The first, proximal part gives rise to a single branch, the superior thoracic artery. The second, central part gives rise to the thoracoacromial artery medially and the lateral thoracic artery laterally. The third, distal part gives rise to three branches, including the subscapular artery, which subdivides into the thoracodorsal and circumflex scapular arteries, and the anterior and posterior circumflex humeral arteries. The axillary vein runs from the upper margin of the teres major muscle to the lateral margin of the first rib, where it becomes the subclavian vein. The axillary vein is formed from the confluence of the basilic, brachial and cephalic veins. The axillary venous tributaries follow the corresponding arterial branches and are named accordingly.

**Figure 21:**
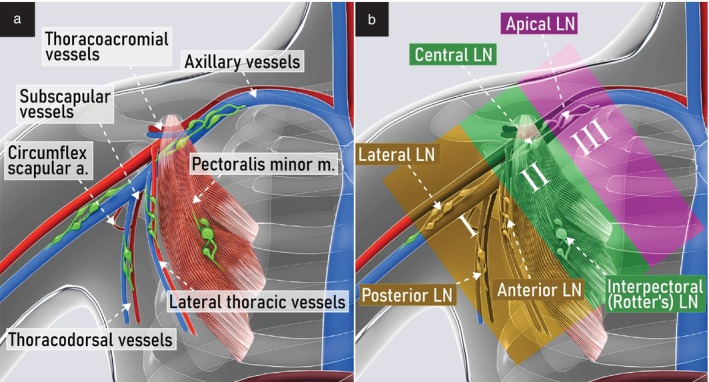
Axillary lymph nodes. Schematic diagram showing essential ultrasound landmarks. (a) Main anatomical landmarks of the axillary region. (b) Location of the five groups of axillary lymph nodes and their respective levels. Interpectoral (Rotter's) lymph nodes are also located at the center of the pyramid, but more superficially. a., artery; LN, lymph node(s); m., muscle. (See also Videoclip S13.)

The axillary lymph nodes are distributed along the main vessels and can be found at the basal vertices, middle area and apex of the truncated trapezoid‐based pyramid (Figures 20 and 21). Five lymph‐node groups can be distinguished in a distal to proximal direction, as follows. The lateral (humeral) lymph nodes are found along the distal part of the axillary artery. The posterior (subscapular) lymph nodes are located along the subscapular and thoracodorsal vessels. The anterior (pectoral) lymph nodes are found at the trapezoid base, along the lateral thoracic vessels. The central group is located at the center of the pyramid, along the middle part of the axillary artery. Also at the center of the pyramid, the interpectoral lymph nodes (Rotter's nodes) can be found, in the layer between the pectoralis major and minor muscles^31^. Finally, the apical group is located at the apex of the pyramid, along the proximal part of the axillary artery^11^, ^32^.

Additionally, the axillary lymph nodes can also be subdivided into three levels, defined according to their relation with the pectoralis minor muscle, which is the reference landmark. The pectoralis minor muscle runs obliquely from the third to fifth ribs to its attachment to the coracoid process of the scapula. The first level includes the lymph nodes lateral to the pectoralis minor, the second level includes those between its lateral and medial borders, and the third level includes those medial to it (Figure 21)^33^.

#### Examination technique

A high‐frequency linear‐array transducer is preferred, although a lower‐frequency convex‐array transducer can be used in obese patients. The patient lies in a supine position, with her arm abducted and extrarotated, with her hands lying behind her head (Videoclip S13)^34^.

**Figure 22:**
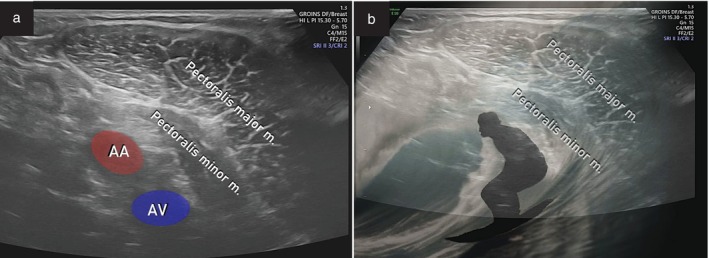
Surfer sign. Images illustrating the ultrasound appearance of this novel sign, obtained using a transcutaneous approach with a linear‐array probe (7.5–15 MHz, transverse plane). The axillary artery (AA) represents the surfer's head, the axillary vein (AV) is his bottom, and the pectoralis muscles form the wave tunnel behind him. m., muscle. (See also Videoclip S13.)

The base of the axillary pyramid is identified as the space between the anterior and posterior axillary folds. The examination begins by placing the probe in transverse section at the caudal and lateral edge of the pectoralis major muscle. The probe is moved upwards to the axillary vessels, which are visualized in their cross‐section. By sliding the probe along the anterior wall, the pectoralis major and minor muscles are followed with the axillary vessels below, like a surfer in a wave tunnel. The ‘surfer sign’ indicates the intersection of the first and second levels of the axillary lymph nodes (Figure 22).

**Figure 23:**
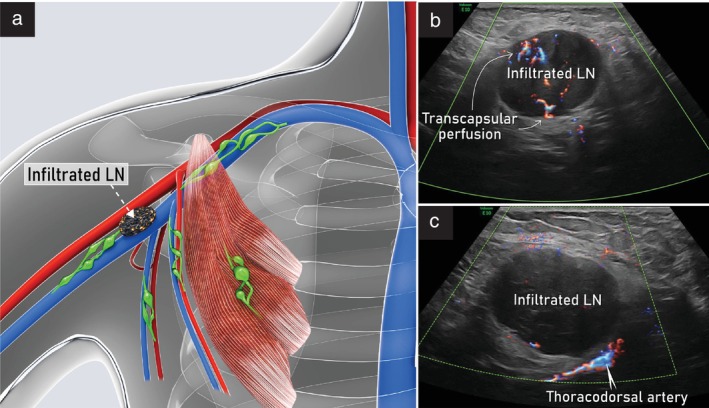
Pathological axillary lymph node. (a) Schematic diagram. (b,c) Appearance on ultrasound with power Doppler assessment of infiltrated axillary lymph node found at Level I (as defined in Figure 21) along the thoracodorsal vessels, obtained with a linear‐array probe (7.5–15 MHz) positioned in a transverse plane. Note presence of transcapsular flow (b) in the infiltrated lymph node. Corresponding clinical case is presented in Videoclip S14. LN, lymph node.

Rotating the probe parallel to the anterior fold and moving laterally, the lymph nodes of the first level can be assessed. Most axillary lymph nodes are located here, where they are always visible (Figure 23). Caudally, the crest of the lateral margin of the scapula and the muscles of the posterior wall, including latissimus dorsi, teres major and subscapularis muscles, are visualized. More cranially, the muscles of the lateral wall can also be observed. The scan continues going back over the muscles of the anterior wall, exploring the area under the belly of the pectoralis minor, with the probe aligned with the longitudinal axes of the axillary vessels. Thereby, the second lymph node level can be explored fully, keeping the probe perpendicular to the skin plane and making small upward‐and‐downward oscillatory movements above the vessels. Both the lymph nodes below the pectoralis minor and Rotter's interpectoral lymph nodes are difficult to recognize at this level unless strongly reactive or metastatic. The transition to the third level is marked by the medial border of the pectoralis minor muscle. In the proximity of this margin, it is also possible to visualize the emergence of the thoracoacromial vessels, which represent a further transition landmark. Finally, in the third level, the perivascular space is very narrow and lymph nodes are visible only when they are pathological. Examples of abnormal ultrasound findings related to the axillary lymph nodes are shown in Videoclip S14.

### Parasternal (internal mammary/thoracic) lymph nodes

The parasternal (internal mammary/thoracic) lymph nodes drain the breasts, the deeper structures of the anterior chest wall, the deeper structures of the anterosuperior abdominal wall and the anterosuperior surface of the liver (through the superior diaphragmatic/cardiophrenic lymph nodes, as discussed above)^11^. Infiltration of the parasternal lymph nodes in gynecological cancer can be via lymph vessels along the inferior epigastric vessels. These vessels anastomose with the superior epigastric vessels, which are the terminal branches of the internal thoracic vessels. Parasternal lymph‐node involvement in gynecological tumors is rare and occurs mainly in recurrent and/or advanced tubo‐ovarian carcinoma with peritoneal disemination^35^.

Several variations of nomenclature can be found in the literature to define this group of lymph nodes. Mostly they are referred to as ‘internal mammary’, but the term ‘internal thoracic’ is also commonly used. According to anatomical terminology, the correct term to describe this group is ‘parasternal lymph nodes’; this is, therefore, the nomenclature of choice and should be used preferentially^7^.

#### Anatomical classification of parasternal lymph nodes

**Figure 24:**
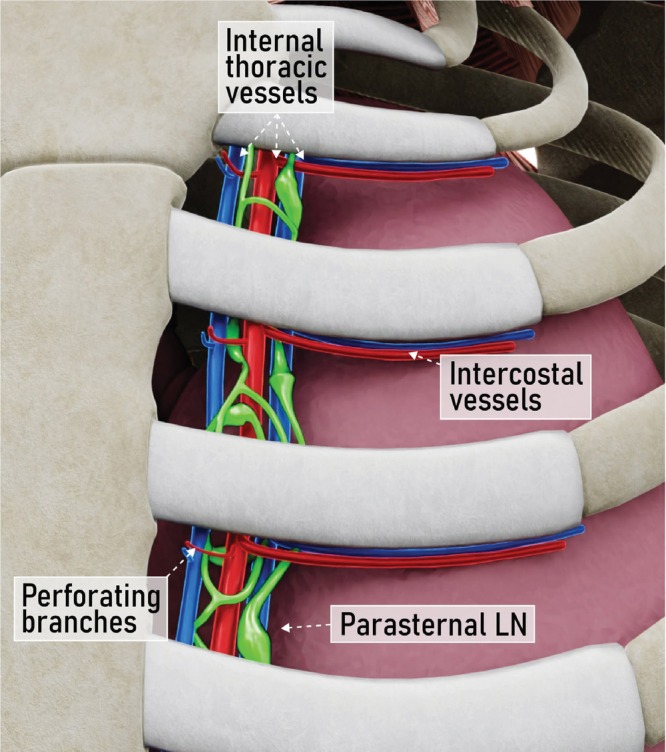
Parasternal lymph nodes. Schematic diagram showing relevant vessels of this region, including internal thoracic vessels, giving rise to the perforating branches for muscular supply. LN, lymph nodes(s). (See also Videoclip S15.)

Parasternal lymph nodes are located mainly in the first five, and most frequently in the second, third and fourth, intercostal spaces^36^, ^37^. They are not always visible on ultrasound, but can become apparent when they are inflamed or pathological^36^, ^37^. They are located anterolaterally to the internal mammary/thoracic vessels (Figure 24). The internal mammary artery originates from the prescalene part of the subclavian artery, approximately 2 cm from the sternal head of the clavicle. It runs caudally in the intercostal spaces, about 1 cm from the lateral face of the sternum, on the posterior aspect of the costal cartilage, and is separated from the pleura by the endothoracic fascia and the transversus thoracis muscle. It gives rise to the sternal, anterior intercostal and perforating branches, and then bifurcates into its terminal branches, the musculophrenic artery and the superior epigastric artery. The medial and lateral internal thoracic veins run alongside the artery, converging medial to it at the level of the third costal cartilage, forming a single internal thoracic vein before draining into the brachiocephalic vein on each side.

The parasternal lymph nodes drain into the bronchomediastinal lymphatic trunk, which on the left drains into the thoracic duct and on the right drains into the right lymphatic duct.

#### Examination technique

A high‐frequency linear‐array transducer is preferred, although a lower‐frequency convex‐array transducer can be used in obese patients (Videoclip S15). The patient lies in a supine position, with her arm either abducted and externally rotated, with hands lying behind the head, or ideally with her arm in a neutral position alongside the torso^32^.

The probe is placed at the lateral border of the manubrium of the sternum in a longitudinal view, close to the first rib. Moving caudally along the intercostal spaces, the ribs, intercostal muscles and internal mammary vessels are visualized. Within each intercostal space, small movements back and forth in a medial‐to‐lateral direction can be made to visualize the internal mammary artery and vein. The endothoracic fascia appears as a hyperechogenic line underneath these structures and separates them from the lung. Perforating branches can be observed arising from the internal mammary vessels and reaching the overlying muscles.

The scan is then repeated in the transverse view to identify the same structures, offering clearer visualization of these lymph nodes compared with the longitudinal view. Lymph nodes are not always identified but may typically be found along the vessels in the virtual space between the intercostal muscle ventrally and the endothoracic fascia dorsally.

## CONCLUSION

Recent advances in ultrasound technology and the use of combined sonographic approaches allow accurate evaluation of lymph‐node involvement in advanced gynecological cancer, based on the identification of changes in the internal architecture and vascular patterns in lymph nodes according to VITA terminology. Both infradiaphragmatic and supradiaphragmatic regions can now be evaluated. Prerequisites for effective ultrasound evaluation include a sound knowledge of the relevant anatomy and lymphatic drainage pathways, as highlighted in this consensus opinion, the use of a systematic approach in combination with structured sonographic training in a specialized gynecological oncology center, the availability of state‐of‐the‐art equipment and appropriate acoustic conditions. In the hands of an operator with expertise in gynecological cancer diagnosis and treatment, who always takes into account the individual clinical scenario and risk factors for lymph‐node involvement, ultrasound represents a highly effective tool with which to identify potentially infiltrated lymph nodes in a patient presenting with gynecological cancer.

## Supporting information


**Figure S1** Schematic diagrams showing measurements and terminology to be used to describe lymph nodes according to the Vulvar International Tumor Analysis (VITA) consensus opinion on terms, definitions and measurements to describe sonographic features of lymph nodes (reproduced from Fischerova *et al*.^5^).

**Table S1** Ultrasound parameters for description of lymph‐node evaluation according to the Vulvar International Tumor Analysis (VITA) consensus opinion^5^

**Videoclip S1** Clinical cases: pelvic parietal lymph nodes.

**Videoclip S2** Methodology: abdominal lymph nodes.

**Videoclip S3** Clinical cases: abdominal parietal lymph nodes.

**Videoclip S4** Clinical cases: pelvic visceral lymph nodes.

**Videoclip S5** Clinical cases: abdominal visceral lymph nodes.

**Videoclip S6** Methodology: inguinal lymph nodes.

**Videoclip S7** Clinical cases: inguinal lymph nodes.

**Videoclip S8** Methodology: superior diaphragmatic lymph nodes.

**Videoclip S9** Clinical cases: superior diaphragmatic lymph nodes.

**Videoclip S10** Methodology: supraclavicular lymph nodes.

**Videoclip S11** Clinical cases: supraclavicular lymph nodes.

**Videoclip S12** Anatomy: axilla.

**Videoclip S13** Methodology: axillary lymph nodes.

**Videoclip S14** Clinical cases: axillary lymph nodes.

**Videoclip S15** Methodology: parasternal lymph nodes.

## Data Availability

Data sharing is not applicable to this article as no new data were created or analyzed in this study.
