# Supplementary material for: Ultrasound assessment of lymph nodes for staging of gynecological cancer: consensus opinion on terminology and examination technique
Source: Ultrasound Obstet Gynecol. 2024 Nov 8;65(2):206–25. doi: 10.1002/uog.29127 (PMC12133214; doi:10.1002/uog.29127)
Supplement: Supplementary file 1 — Figure S1 Schematic diagrams showing measurements and terminology to be used to describe lymph nodes according to the Vulvar International Tumor Analysis (VITA) consensus opinion on terms, definitions and measurements to describe sonographic features of lymph nodes (reproduced from Fischerova et al. 5 ). [file UOG-65--s012.docx]

**Figure S1** Schematic diagram showing the measurements and terminology to be used to describe lymph nodes according to the VITA consensus. VITA, (Vulvar International Tumor Analysis consensus opinion on terms, definitions and measurements to describe sonographic features of lymph nodes.^1^


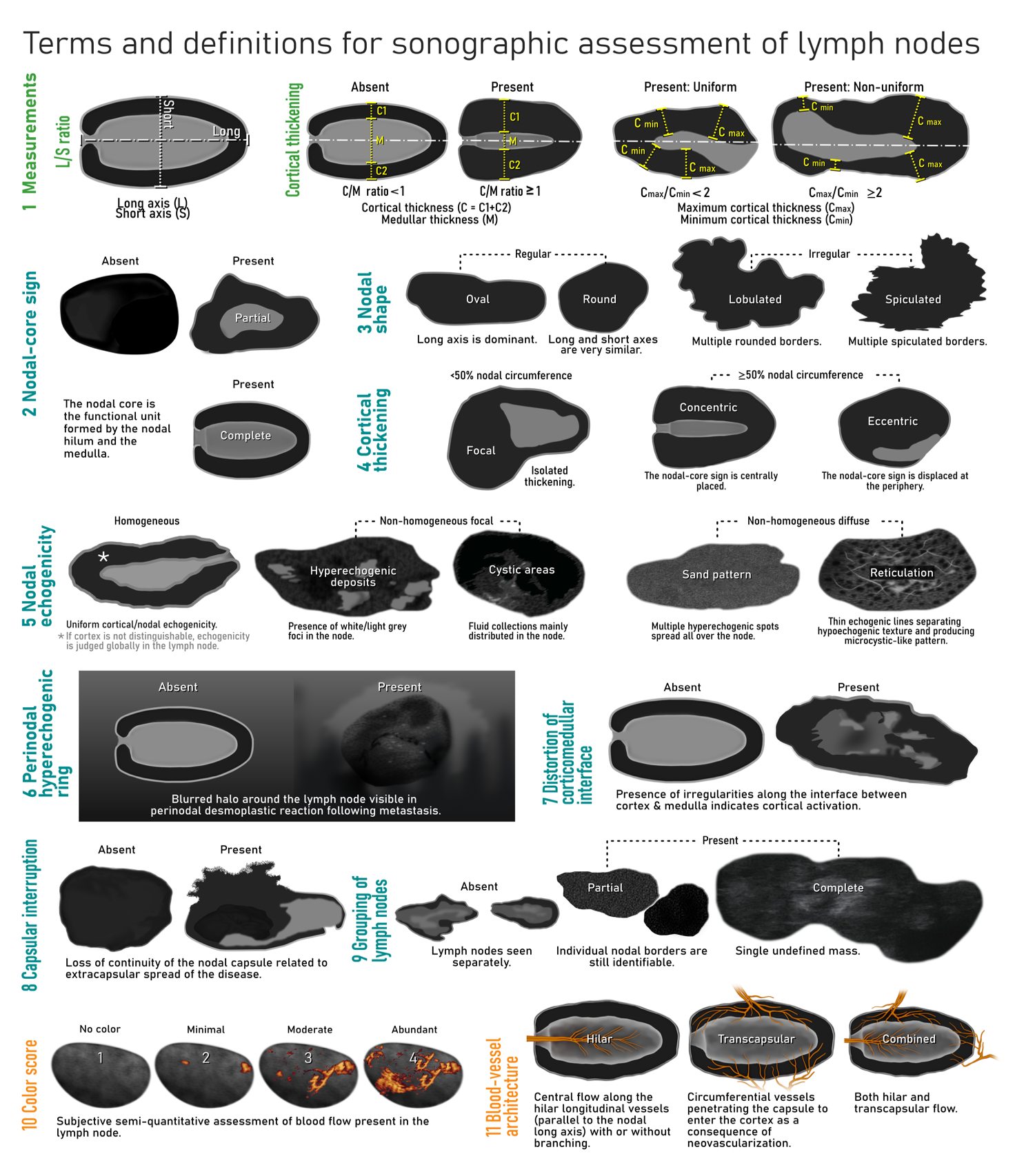


**Reference**

1. Fischerova D, Garganese G, Reina H, et al. Terms, definitions and measurements to describe sonographic features of lymph nodes: consensus opinion from the Vulvar International Tumor Analysis (VITA) group. *Ultrasound Obstet Gynecol*. Jun 2021;57(6):861-879. doi:10.1002/uog.23617
