# Supplementary material for: Ultrasound assessment of lymph nodes for staging of gynecological cancer: consensus opinion on terminology and examination technique
Source: Ultrasound Obstet Gynecol. 2024 Nov 8;65(2):206–25. doi: 10.1002/uog.29127 (PMC12133214; doi:10.1002/uog.29127)
Supplement: Supplementary file 2 — Table S1 Ultrasound parameters for description of lymph‐node evaluation according to the Vulvar International Tumor Analysis (VITA) consensus opinion 5 [file UOG-65--s008.docx]

**Table S1** Ultrasound parameters for description of lymph-node evaluation according to the Vulvar International Tumor Analysis (VITA) consensus opinion

| **Ultrasound parameter** | **Description** | |
| --- | --- | --- |
| **Lymph-node dimensions**  *Quantitative assessment* | - Long axis (longest diameter of the lymph node in any plane) - Short axis (maximum diameter of the lymph node perpendicular to its long axis) - Lymph-node shape (L/S ratio): L/S ratio ≥ 2 » benign; L/S ratio < 2 » malignant - Cortical thickening (C/M ratio – at the widest point of cortical thickening perpendicular to the long axis of the lymph node); cortical thickening = C/M ratio ≥ 1 - Uniformity of cortical thickening (Cmax/Cmin). Uniform cortical thickening: Cmax/Cmin < 2; non-uniform cortical thickening: Cmax/Cmin ≥ 2 on either or both sides of the lymph node | |
| **Morphological assessment**  *Qualitative assessment* | Nodal shape (regular: round, oval; irregular: lobulated, spiculated) | |
|  | Nodal-core sign | **Present**   - Complete: hilum and medulla visible - Partial: medulla visible but hilum not detectable   **Absent**: hilum and medulla not detectable |
|  | Cortical thickening | |
|  | Nodal echogenicity | |
|  | Capsular interruption | |
|  | Distortion of corticomedullar interface | |
|  | Perinodal hyperechogenic ring | |
|  | Grouping of lymph nodes (matting) | |
| **Vascularization assessment** | Blood-vessel architecture | |
|  | Color score* | |
| **Lymph-node status** | **Assessment by standardized VITA terminology using the classification LN1 – LN5:**   - LN1: Normal finding - LN2: Benign finding - LN3: Indeterminate, probably benign finding - LN4: Probably malignant finding - LN5: Malignant finding | |

*Color score following International Ovarian Tumour Analysis (IOTA) terms and definitions (color score 1, no perfusion; color score 2, minimal perfusion; color score 3, moderate flow; color score 4, highly vascularized). C/M ratio, cortex/medulla ratio; Cmax/Cmin, maximum cortical thickening/minimum cortical thickening; L/S ratio, long-/short-axis ratio.

**Reference**

Fischerova D, Garganese G, Reina H, et al. Terms, definitions and measurements to describe sonographic features of lymph nodes: consensus opinion from the Vulvar International Tumor Analysis (VITA) group. *Ultrasound Obstet Gynecol*. Jun 2021;57(6):861-879. doi:10.1002/uog.23617
